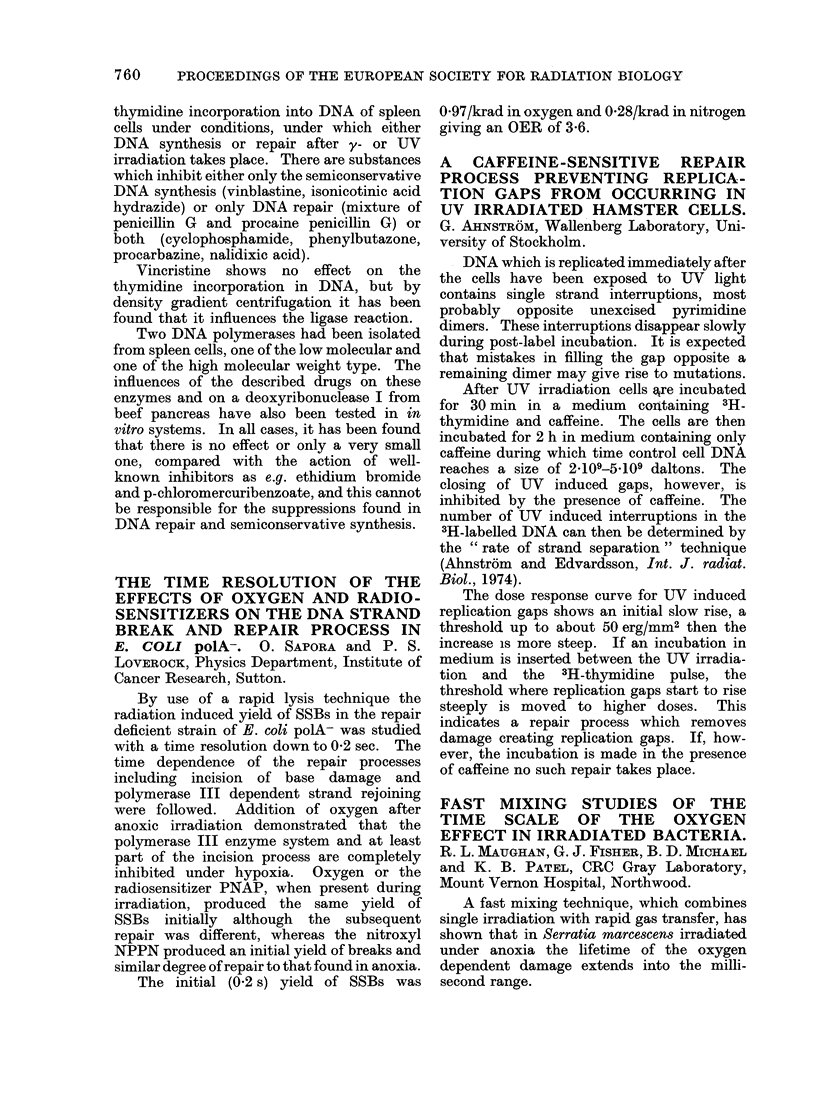# Proceedings: The time resolution of the effects of oxygen and radio-sensitizers on the DNA strand break and repair process in E. coli polA.

**DOI:** 10.1038/bjc.1975.319

**Published:** 1975-12

**Authors:** O. Sapora, P. S. Loverock


### THE TIME RESOLUTION OF THE EFFECTS OF OXYGEN AND RADIO-SENSITIZERS ON THE DNA STRAND BREAK AND REPAIR PROCESS IN *E. COLI* polA⁻.

O. Sapora and P. S. Loverock, Physics Department, Institute of Cancer Research, Sutton.

By use of a rapid lysis technique the radiation induced yield of SSBs in the repair deficient strain of *E. coli* polA$^-$ was studied with a time resolution down to 0·2 sec. The time dependence of the repair processes including incision of base damage and polymerase III dependent strand rejoining were followed. Addition of oxygen after anoxic irradiation demonstrated that the polymerase III enzyme system and at least part of the incision process are completely inhibited under hypoxia. Oxygen or the radiosensitizer PNAP, when present during irradiation, produced the same yield of SSBs initially although the subsequent repair was different, whereas the nitroxyl NPPN produced an initial yield of breaks and similar degree of repair to that found in anoxia.

The initial (0·2 s) yield of SSBs was 0·97/krad in oxygen and 0·28/krad in nitrogen giving an OER of 3·6.